# Endocavitary contrast enhanced ultrasound (CEUS): a novel problem solving technique

**DOI:** 10.1007/s13244-018-0601-x

**Published:** 2018-03-28

**Authors:** G. T. Yusuf, C. Fang, D. Y. Huang, M. E. Sellars, A. Deganello, P. S. Sidhu

**Affiliations:** Department of Radiology, King’s College Hospital, Denmark Hill, London, SE5 9RS UK

**Keywords:** Ultrasound imaging, Contrast agents, Microbubbles, Diagnostic ultrasound, Ultrasound, interventional

## Abstract

**Abstract:**

Contrast-enhanced ultrasound (CEUS) is a technique that has developed as an adjunct to conventional ultrasound. CEUS offers a number of benefits over conventional axial imaging with computerised tomography and magnetic resonance imaging, primarily as a “beside” test, without ionising radiation or the safety concerns associated with iodinated/gadolinium-based contrast agents. Intravascular use of ultrasound contrast agents (UCAs) is widespread with extensive evidence for effective use. Despite this, the potential utility of UCAs in physiological and non-physiological cavities has not been fully explored. The possibilities for endocavitary uses of CEUS are described in this review based on a single-centre experience including CEUS technique and utility in confirming drain placement, as well as within the biliary system, urinary system, gastrointestinal tract and intravascular catheters.

**Teaching Points:**

*• CEUS offers an excellent safety profile, spatial resolution and is radiation free.*

*• Endocavitary CEUS provides real-time imaging similar to fluoroscopy in a portable setting.*

*• Endocavitary CEUS can define internal architecture of physiological cavities.*

*• Endocavitary CEUS can confirm drain position in physiological and non-physiological cavities*.

## Introduction

Contrast-enhanced ultrasound (CEUS) has developed as a technique with increasing acceptance in recent years. Although the most widespread use of CEUS has been in the characterisation of focal liver lesions [1], many other uses have been developed including renal, aortic, bowel and testicular evaluation [2]. CEUS is expected to gain further popularity following the recent Food and Drug Administration approval in the USA of Lumason™/SonoVue™ (Bracco, Milan) for hepatic and paediatric hepatic use [3]. The rapidly increasing myriad uses of CEUS have led to the development of guidelines, issued by the European Federation and Society of Ultrasound in Medicine and Biology (EFSUMB) examining both hepatic and non-hepatic applications [1, 2]. These guidelines are based almost entirely on conventional intravascular use. The EFSUMB guidelines do acknowledge the use of ultrasound contrast agents (UCAs) in the assessment of non-physiological cavities and state that in theory this should be safe, based on the excellent safety profile from intravascular use in both adults and paediatrics [4, 5]. Despite intra-cavitary usage being reported, the EFSUMB refrains from making recommendations other than use on a case-by-case basis given the lack of experience in this area [2].

CEUS is an adjunctive technique to conventional B-mode and Doppler ultrasound (US), giving rise to the term multiparametric ultrasound (MPUS) [6]. The microbubble ultrasound contrast agent (UCA) most often used in Europe, SonoVue™, consists of sulphur hexafluoride, an inert gas, enveloped in a phospholipid shell and is a truly intravascular agent with pulmonary excretion of the gas and hepatic clearance of the phospholipid shell [7]. At low acoustic pressure microbubbles resonate, giving rise to non-linear signals, which are detected by modifications of the conventional ultrasound machine. Following cancellation of the linear signal from normal tissue, the non-linear signal provides a contrast image with dynamic assessment on a macro- and microvascular scale when used intravenously. These properties make CEUS appropriate for endocavitary use, with excellent spatial and temporal resolution in real time, akin to fluoroscopic imaging but without the need for iodinated contrast or ionising radiation. There are also the traditional advantages of US imaging, including the ability to perform imaging at the bedside, in immobile patients, particularly important in an intensive care setting. As a result CEUS offers an alternative means of dynamic imaging in the population potentially at increased risk from conventional means such as paediatric patients, renal failure patients and those for whom transport to a radiology department would be impractical or unsafe. Many individual case reports and case series on the utilisation of endocavitary CEUS exist [8, 9], but there is no unifying technique or definitive list of applications. Generally endocavitary CEUS is used for confirming catheter drain placement, tracking the course of a physiological or non-physiological cavity, identifying filling defects and the presence of fistulation. This review article aims to describe the myriad uses implemented in a single centre and to consider the recent literature on the utility of endocavitary CEUS.

## Endocavitary CEUS technique

Endocavitary CEUS is a developing technique and a methodology for general use remains undefined. Conventionally intravenous use of CEUS requires 1.2 ml – 4.8 ml of SonoVue™ depending on the organ or site examined. Endocavitary CEUS differs in that the volume of the solvent, the fluid within a cavity, is much smaller than the volume of circulating blood in the vascular tree, and a considerably lower dose of UCA is required. In vitro studies have shown SonoVue™ to be stable in a diluted state providing sufficient backscatter for endocavitary use [10]. In addition, the lack of circulation of the UCA results in a greater degree of stability, with the UCA estimated to remain within a collection for up to 20–30 min [10, 11]. Prior feasibility studies and case reports suggest 0.1 ml SonoVue™ diluted in 10-20 ml 0.9 % saline is sufficient for intra-cavitary use, whilst for oral administration 1 drop of the UCA is diluted in 50 ml water [11]. The authors use a dilution of 0.1 ml SonoVue™ in 50 ml 0.9 % saline routinely. The administered volume of the solution varies on a case-by-case basis, as high-frequency linear transducers may require higher concentrations of UCA, given the optimal imaging for CEUS is 2–3 MHz [12]. For studies of the gastrointestinal tract where a larger volume would be potentially needed, the ratio can be extrapolated in up to 200 ml of solution.

Adverse reactions to intravenous CEUS have been rarely described and UCAs have been shown to have an excellent safety profile [4, 5]. Due to the absence of contact with the systemic circulation, endocavitary CEUS is expected to have a lower rate of adverse incidents, but this has not yet been proven and access to resuscitation equipment must be considered essential. Contraindications to endocavitary CEUS are not yet available given that it is a technique in its infancy. Contraindications to intravenous CEUS are severe cardiopulmonary dysfunction and known hypersensitivity, whilst it has been deemed preferable to avoid intravenous CEUS in pregnancy because of an absence of clinical data.

Endocavitary CEUS is performed following optimised greyscale and Doppler imaging using contrast-specific software with a low mechanical index (< 0.2). Either high frequency linear transducers or a low frequency curvilinear transducer can be used for imaging with no adjustment of contrast volume or concentration required.

## Endocavitary uses

### Intrauterine

**Fig. 1:**
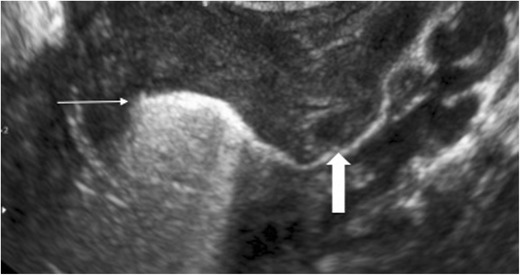
CEUS hysterosalpingogram. Transvaginal ultrasound following intrauterine administration of the ultrasound contrast agent. Contrast fills the endometrial cavity (thin arrow) and unobstructed flow is seen along the left fallopian tube confirming patency (thick arrow). (Image courtesy of Dr. Luca Savelli, Bologna, Italy)

One of the earliest recognised uses for endocavitary CEUS was for evaluation of fallopian tube patency [13] (Fig. 1) and this has been recognised by the National Institute of Clinical Excellence (UK), leading its incorporation as an alternative to the conventional fluoroscopic hysterosalpingogram (HSG). This has led to a reduction in the implementation of diagnostic laparoscopy and more efficient use of resources [14, 15]. A 100 % concordance has been found with traditional fluoroscopic imaging with 97 % specificity and 82 % sensitivity [16] whilst conferring no difference in patient discomfort. An alternative to the traditional HSG is hysterosalpingosonography (HSS) conventionally performed using agitated saline or air. However, substituting an ultrasound contrast agent resulted in an increase in diagnostic accuracy from 77 % to 85 % [13] by allowing clear delineation of the uterine cavity and free flow of contrast along the Fallopian tubes and peritoneal spill. A disadvantage of hysterosalpingo-contrast sonography (HSCS) is the associated expense of the contrast agent and the EFSUMB guidelines [2] suggest the use of HSCS over HSS only when the latter has failed.

### Fluid collections

**Fig. 2:**
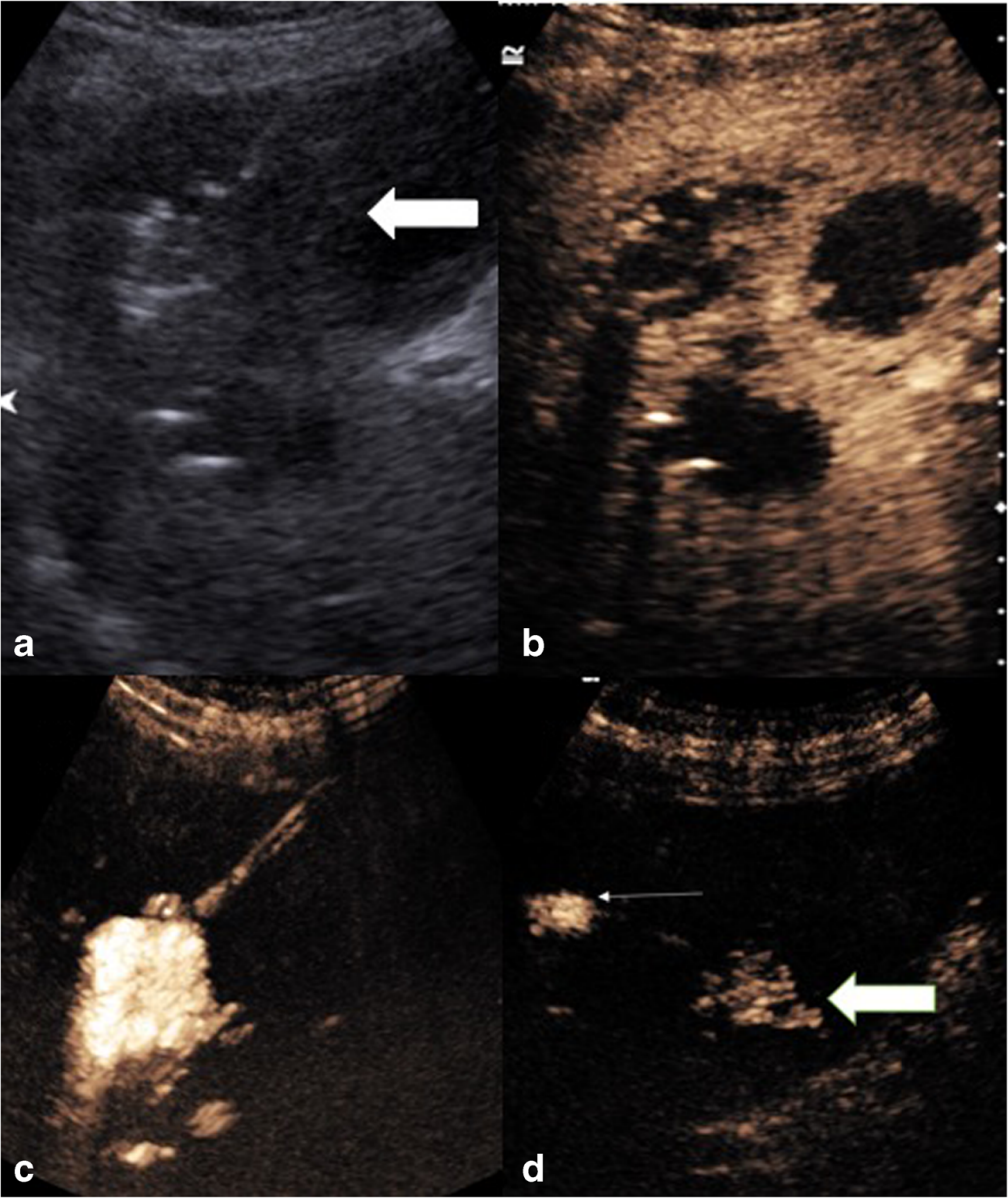
(A) Greyscale imaging of the left lobe of the liver with an ill-defined lesion (large arrow), containing locules of gas. (B) Following administration of intravenous ultrasound contrast the non-enhancing liver abscess with multiple locules is clearly seen. (C and D) Dilute ultrasound contrast injected via an in-dwelling catheter within one locule, confirms correct placement and confirms connections between multiple locules (thin and thick arrows)

**Fig. 3:**
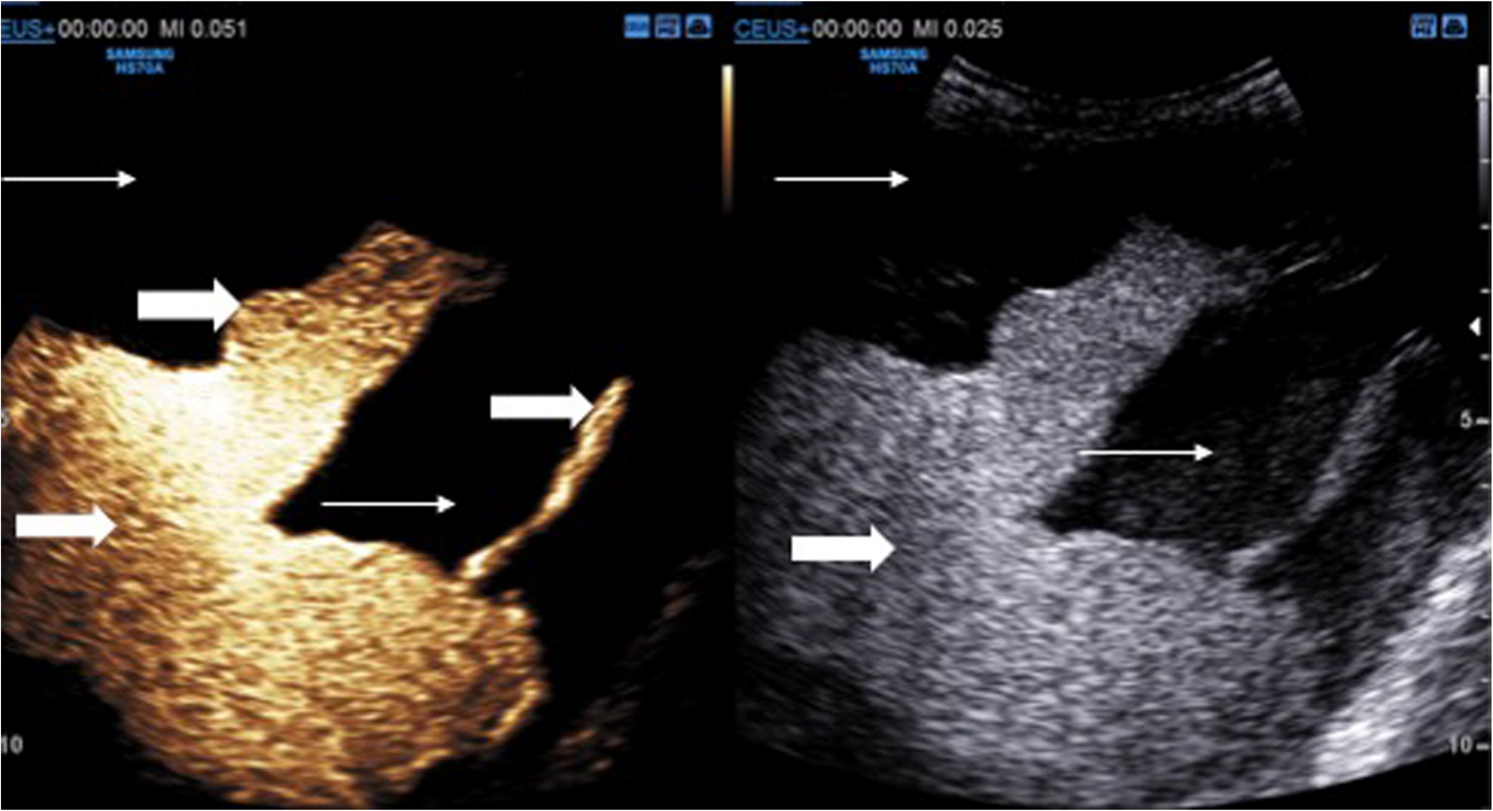
CEUS image following ultrasound contrast administration through the in-dwelling pleural catheter with simultaneous greyscale image. Contrast is confined to a solitary pleural loculation (thick arrows), confirming the drain lies within an isolated locule. The remaining pleural fluid is not opacified and does not communicate with the in-dwelling drain (thin arrows)

Fluid collections are normally readily identifiable by US, with a longstanding established indication for detection and directing intervention. However complications of fluid collections can include difficulty establishing communications between multiple aspects, fistulation, and evaluation of size or confirmation of catheter drain placement. When clinically warranted to evaluate the collection further, contrast-enhanced fluoroscopic imaging may be required or more often static imaging with computed tomography (CT) or magnetic resonance (MR) imaging is needed; conventional US may not be able to provide sufficient information. CEUS often adds another dimension to the baseline US examination. Explorations of endocavitary use of CEUS via in-dwelling pigtail catheter drains including for loculated ascites, pleural drains and liver abscess drains have been performed. Invariably, the drain position can be readily confirmed as the dilute UCA is administered and disperses within the collection but is contained within the loculation of the collection (Fig. 2). The excellent resolution of endocavitary CEUS allows visualisation of solitary microbubbles and can confirm communication between seemingly disconnected elements (Fig. 3) or potentially fistulation. Conversely loculation of the contained elements indicating a solitary catheter drain would be unsuccessful for complete drainage (Fig. 3). As the UCA remains in a non-circulating cavity the contrast persists for up to 20 min allowing time for detailed evaluation [10, 11].

### Biliary tract

**Fig. 4:**
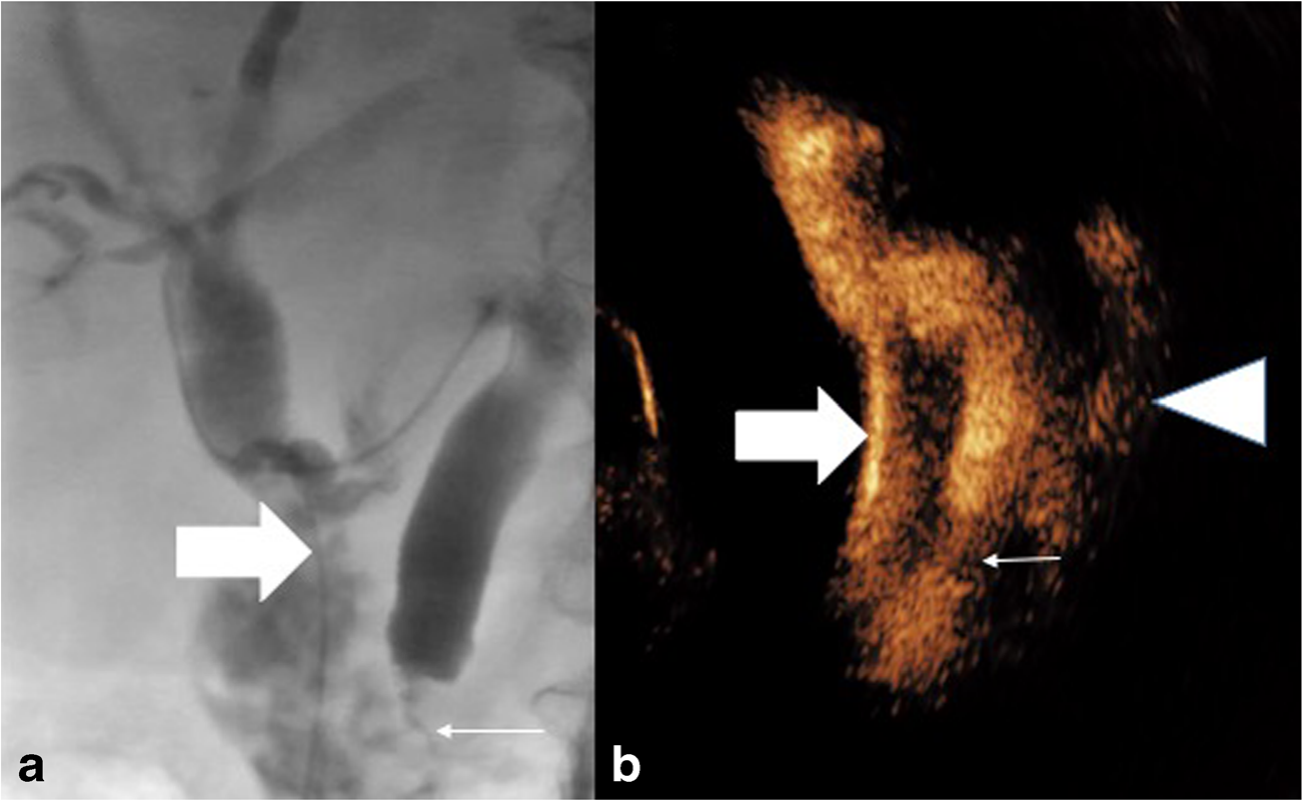
(A) Conventional T-tube cholangiogram under fluoroscopy confirmed a stricture (arrow) but the leak was not seen. (B) Rotated image (for comparative purposes) of a CEUS cholangiogram administered via a T-tube (thick arrow), inserted post cholecystectomy. Ultrasound contrast delineates the common bile duct including a point of stricture (thin arrow) and a contrast extravasation leak (arrowhead)

Intraluminal evaluation of the biliary tree is limited and conventionally is best performed by either magnetic resonance cholangiopancreatography (MRCP) or fluoroscopic cholangiography via a percutaneous approach or a nasobiliary tube. Cholangiography is often performed alongside an intervention such as placement of a biliary drain or stent. Injection of UCA is able to delineate the biliary ducts by opacifiying these ducts with contrast and can potentially identify strictures, leaks or the drain position (Fig. 4). In a study of 12 patients it was suggested that endocavitary CEUS could be used as a bedside tool, but conventional fluoroscopic imaging via T-tube cholangiography was superior [17]. Further studies have shown that intrabiliary endocavitary CEUS is useful for visualisation of an in-dwelling drain tip, with only 54 % seen on conventional US B-mode imaging but 100 % seen with endocavitary CEUS. In addition, intra-biliary CEUS was 96 % accurate for diagnosing a stenotic rather than fully occlusive stricture, corresponding to other studies performed [18]; however abnormalities of the biliary tree are often inadequately visualised, such as irregularities suggestive of sclerosing cholangitis [11]. Daneshi et al. utilised the truly intraluminal nature of UCA to delineate a biliary-vascular fistula as UCA administered via an in-dwelling biliary drain resulted in parenchymal enhancement [8]. Formal studies comparing MRCP to endocavitary CEUS in the biliary system have not been conducted.

### Urinary tract

**Fig. 5:**
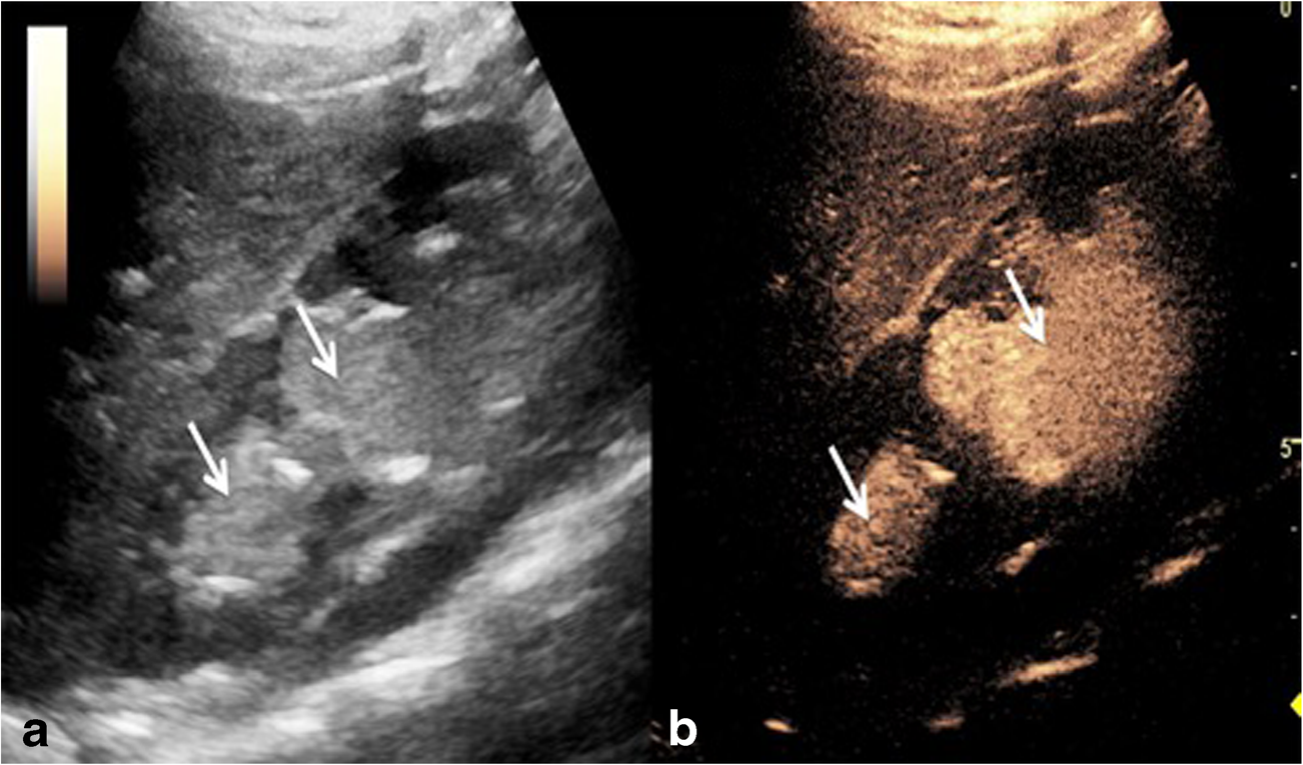
Contrast-enhanced voiding urosonography (CEVUS) with dual screen mode. The right kidney is duplex and is simultaneously demonstrated in (a) greyscale mode and (b) contrast-only mode during bladder filling. Echogenic contrast is seen filling the upper and lower moieties (arrows) of the right kidney retrogradely. There is significant dilation of the renal pelvis and blunting of the renal calyces. These findings are in keeping with grade 3 reflux. (Image courtesy of Dr. Susan Back, Philidelphia, PA, USA)

**Fig. 6:**
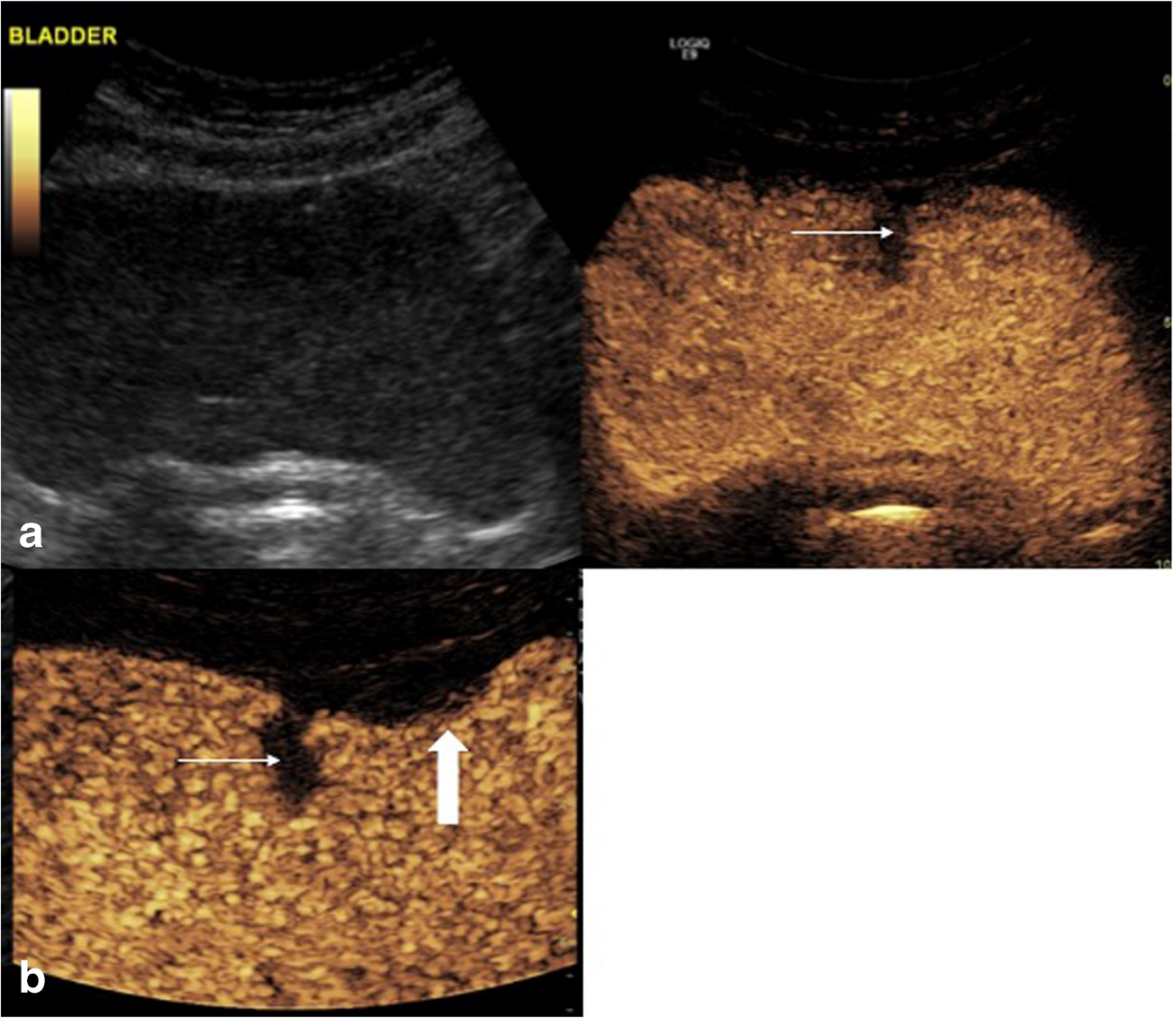
(A) Ultrasound contrast administered via an in-dwelling urinary catheter shows an anterior filling defect seen at cystoscopy to be a villous adenoma within a urachal remnant (thin arrow) with simultaneous greyscale imaging. (B) Optimised CEUS imaging demonstrates the extra vesical element of the filling defect (thick arrow)

Ultrasound contrast agents have been shown to be safe within the urinary collecting system in a number of studies of vesico-ureteric reflux in children replicating the low rate of adverse reactions seen with intravenous use, none of which were classified as any more than minor in nature [19]. Currently identification of vesico-ureteric reflux is the only approved application of endocavitary contrast-enhanced ultrasound, following large trials of safety and diagnostic accuracy [20]. The technique utilises UCA instilled into the bladder and the operator performs sonography in micturition to identify reflux into the ureters and kidneys (Fig. 5). Contrast-enhanced vesico-ureteric reflux studies have been demonstrated to be comparable to conventional fluoroscopic imaging for grading of vesicoureteric reflux in > 90 % of cases [21]. It is accepted that there is a considerable learning curve for this technique and the usual limitations of ultrasound apply, e.g. body habitus, patient compliance. In addition, there is a need to use dilute UCA to prevent acoustic shadowing obscuring the retrovesical ureters, a recognised reason for under-diagnosis of low-grade reflux when using contrast-enhanced voiding ultrasonography. Administration of an UCA via an in-dwelling catheter in adult patients can also allow for bladder assessment. Although no reports exist, endocavitary CEUS cystography may have a potential role in evaluating filling defects (Fig. 6) or possibly evaluation of vesical fistulae.

**Fig. 7:**
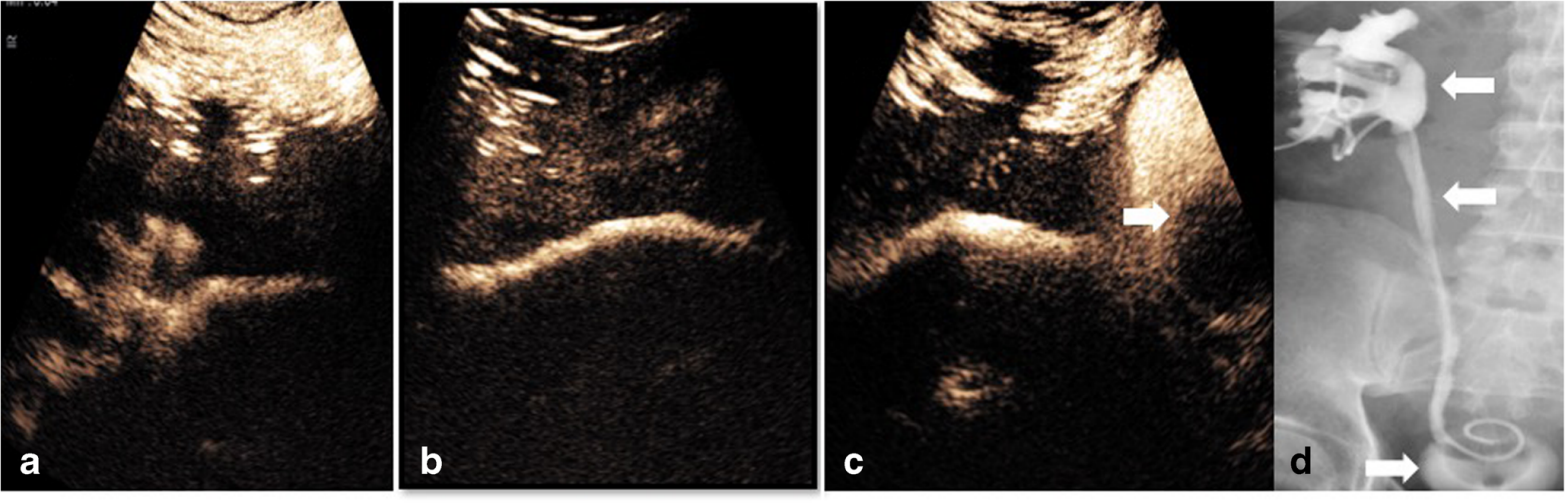
CEUS nephrostogram. Images obtained of the entire ureter and bladder following ultrasound contrast administered through an in-dwelling nephrostomy confirms patency of the collecting system with contrast opacifying the (A) renal calyces, (B) ureter and (C) bladder, which contains a urinary catheter (white arrow). (D) Conventional fluoroscopic nephrostogram confirms free drainage along the collecting system and into the bladder (arrows)

**Fig. 8:**
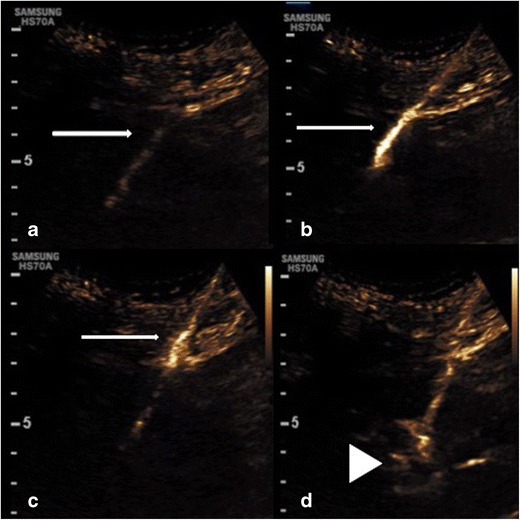
CEUS imaging with ultrasound contrast placed within a Chiba needle. On puncture of a hydro-nephrotic kidney reflux of contrast (A–C, arrow) is shown being ‘pushed’ out by fluid (urine) held under pressure within an obstructed renal pelvis (arrows). Ultrasound contrast is then injected along the Chiba needle to confirm correct needle position and to opacify the renal pelvis (D, arrowhead)

Following percutaneous nephrostomy insertion, it is frequently necessary to assess the renal collecting system and ureter for free passage of contrast prior to removal of the catheter to ensure patency. The stent patency, point of obstruction or relief of a prior obstructing aetiology can all be assessed (Fig. 7); contrast imaging of the ureters also allows for the delineation of a fistula or ureteric rupture. The spatial resolution of endocavitary CEUS allows for a confident assessment of free ureteric drainage. Recent studies demonstrate 79-100% concordance with flouroscopy, with 94% of disconcordant cases showing ureteric patency on CEUS nephrostogram which was not seen on conventional flouroscopy [9, 22]. In an acute setting UCA injected via a needle entering a renal collecting system can be used as an alternative to fluoroscopic imaging enabling confident bedside nephrostomy placement. This percutaneous puncture may be facilitated by placing a small volume of UCA on a puncture needle and watching as the UCA refluxes back along the needle as a calyceal puncture is achieved because of back pressure of urine [23] (Fig. 8).

### Bowel

**Fig. 9:**
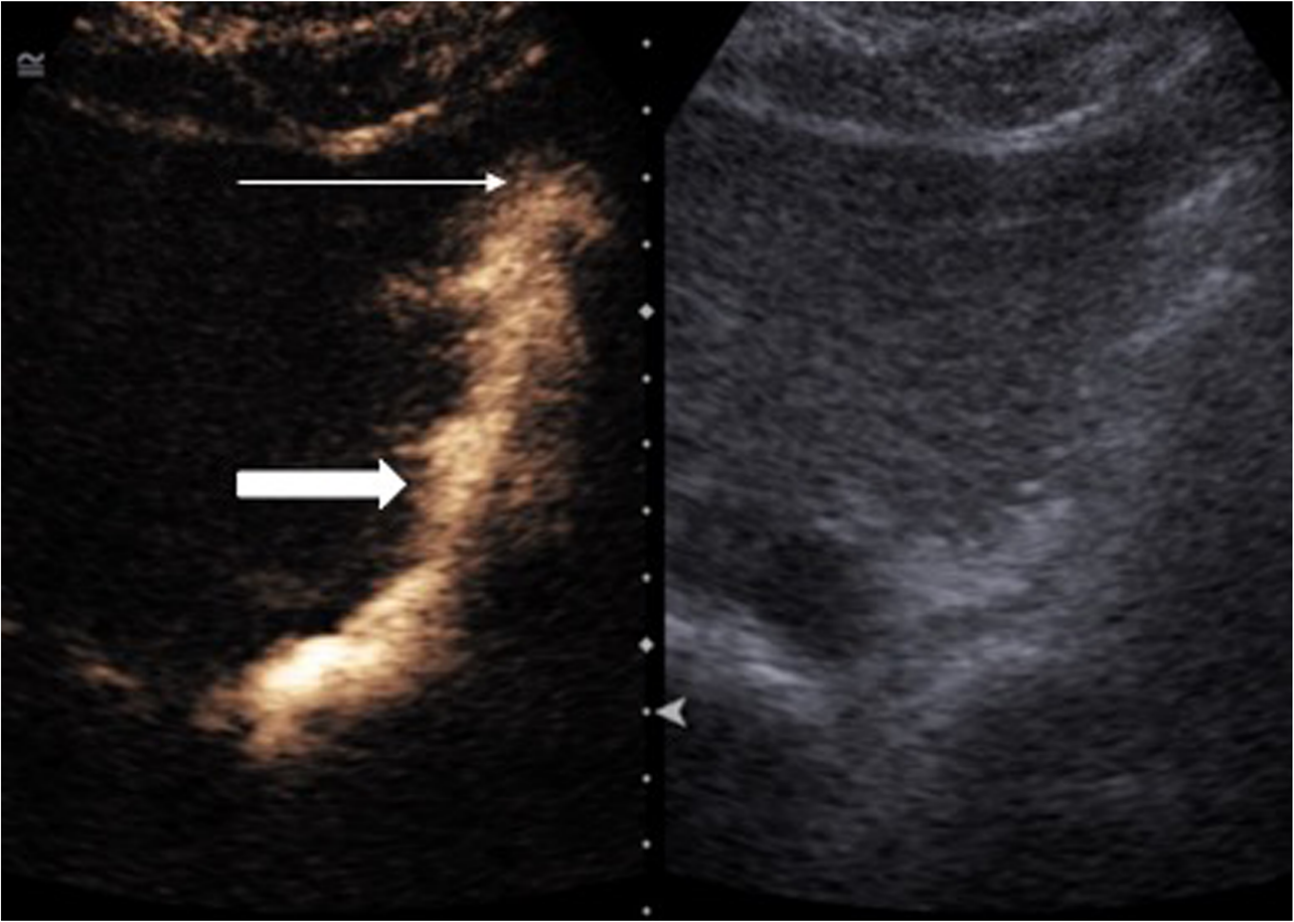
CEUS and corresponding greyscale image following orally administered ultrasound contrast. Distal oesophageal patency is confirmed (arrow) with passage through the gastro-oesophageal junction (thin arrow), with no evidence of a hiatus hernia. Dynamic scanning did not demonstrate gastrointestinal reflux

Trans-abdominal US of the bowel has developed as a technique, and oral solution can be used as a negative contrast agent within the bowel to examine the lumen for evidence of filling defects or complications of inflammatory bowel disease, whilst simultaneous intravenous UCA can allow assessment of the bowel wall [24, 25]. Ultrasound contrast agents have been shown to be stable in the gastric cavity and can be used to allow assessment within the bowel lumen and bowel wall. UCA fills the bowel lumen in a similar way to fluoroscopy, allowing assessment for wall thickening and irregularities; however this may be non-specific particularly in the context of an unprepared bowel. There is a paucity of literature on the use of orally administered UCA, but this has included the assessment of gastrointestinal reflux (Fig. 9), whilst in combination with intravenous CEUS may help in the detection of neoplasia [26]. To date there have been no comparative studies of MR enterography and endocavitary CEUS.

**Fig. 10:**
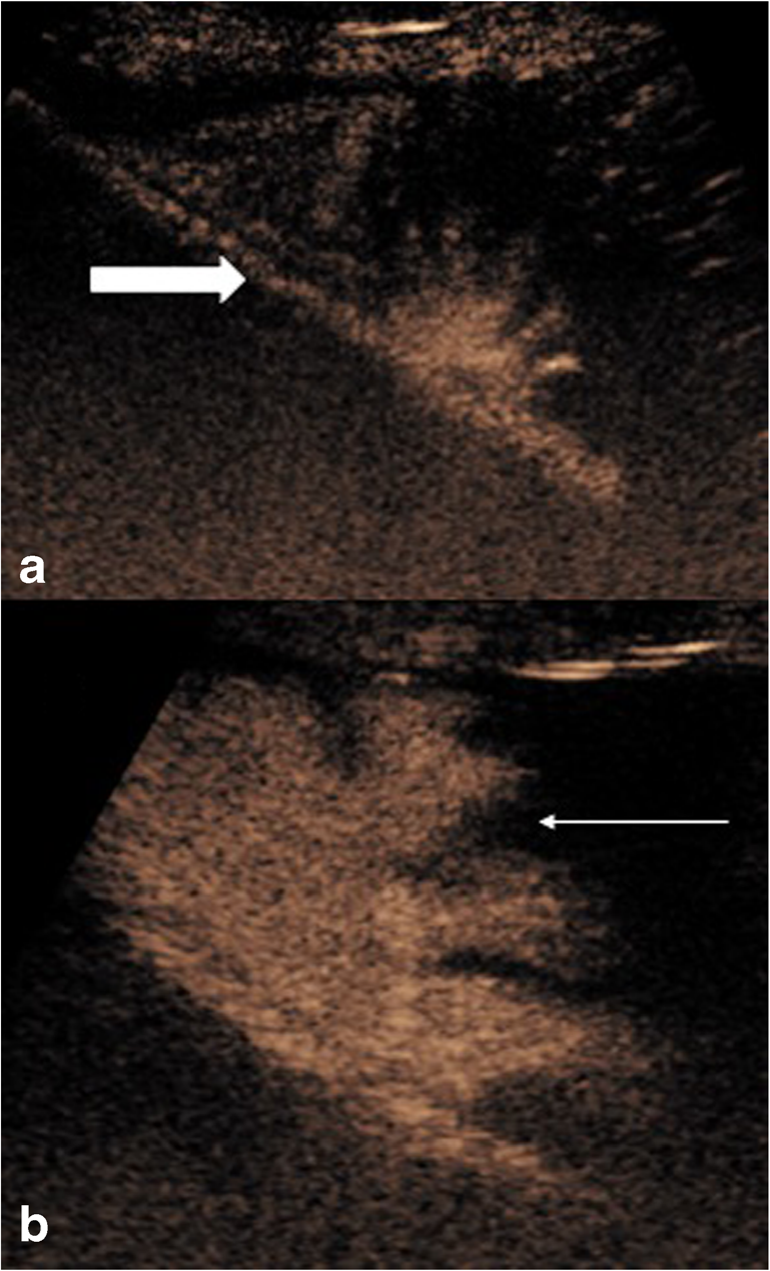
Contrast ultrasound agent administered through a percutaneous jejunostomy (PEJ). (A) Ultrasound contrast instilled via the PEJ tube (thick arrow) confirms correct placement and no evidence of extravasation. (B) A magnified view shows thickening of the small bowel folds (thin arrow)

Gastrostomy and jejunostomy tubes may be placed endoscopically or radiologically, and complications arise, specifically in relation to ‘misplacement’. Even when anchored with a catheter balloon or a “mushroom cage” [27], these tubes can become dislodged in daily activity leading to leakage and infection. Ultrasound contrast agents administered via the tube can confirm correct placement, exclude extravasation and allow assessment of the regional bowel for peristaltic abnormality or wall thickening (Fig. 10).

### Vascular lines

**Fig. 11:**
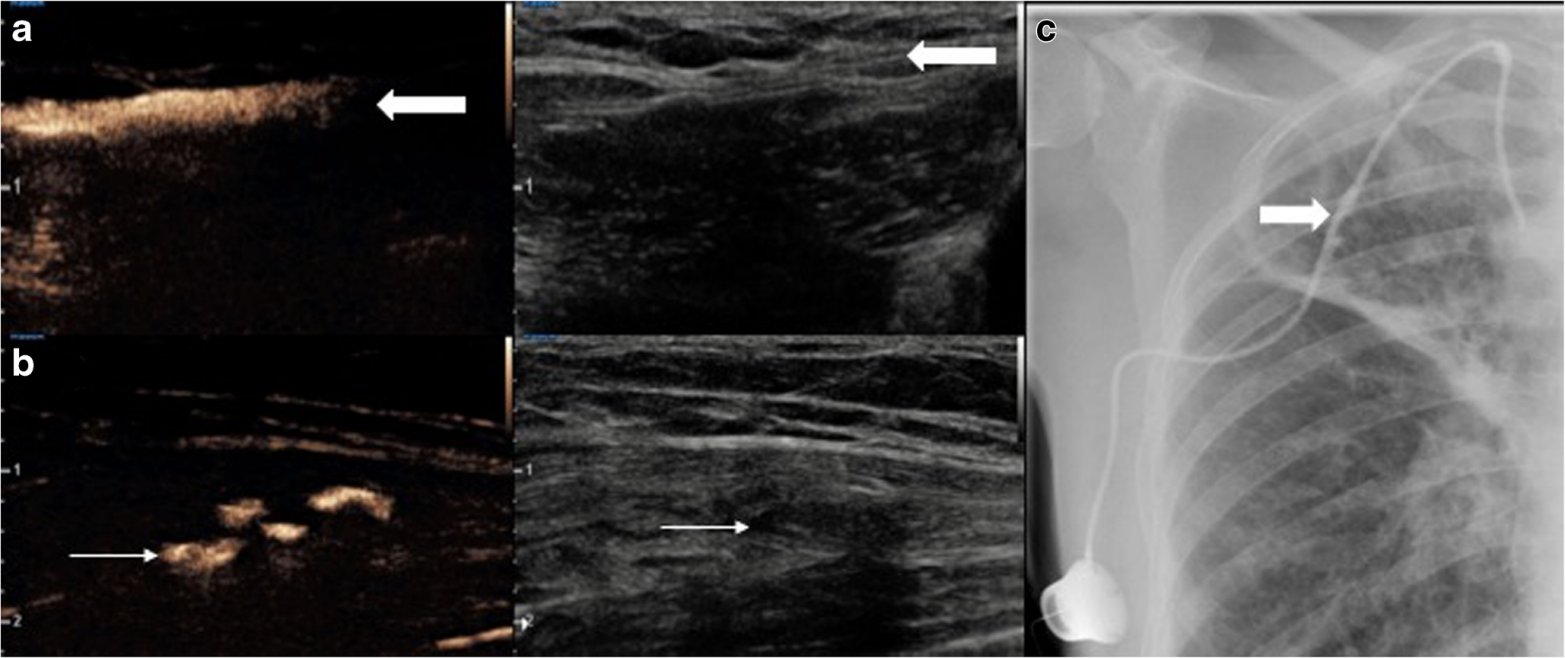
Contrast ultrasound agent administered through a “port-a-cath” for a malfunctioning line causing point tenderness. (A) CEUS and corresponding greyscale image. The subcutaneous course of the line is seen (thick arrow) and luminal patency is confirmed on the contrast image. (B) At the point of tenderness there is contrast extravasation deep to the line where there is a small fluid collection on greyscale imaging (thin arrow). (C) Conventional fluoroscopic linogram, minor irregularity is present along the midportion of the line confirming the site of line rupture (arrow)

Linograms are a common procedure performed in the fluoroscopy suite to ensure an in-dwelling catheter has not become dislodged or potentially damaged (Fig. 11). Most frequently the procedure is performed in patients in whom vascular access is difficult and there is reluctance for line removal such as long-term “porta-caths” or in paedatric patients. Confirmation of a misplaced line warrants line removal, which may involve a general anaesthetic in paediatric patients. Ultrasound contrast agents injected through a line should result in systemic vascular opacification confirming correct placement of the tip, similar to conventional fluoroscopic investigation. Further complications of line placement may also be assessed including the presence of thrombus surrounding a line, as evidenced by lack of vascular enhancement. The lumen of the line itself also often lies superficially and can be assessed for the presence of rupture using a high frequency transducer, particularly important in the presence of focal tenderness (Fig. 11).

### Limitations

Endocavitary CEUS has limitations. Traditional factors that hinder ultrasound remain a factor in endocavitary CEUS and may include body habitus, bowel gas and operator skill. In addition, it is important to use larger volumes of UCA solution in endocavitary use to prevent pooling of contrast, rather than dynamic free diffusion, within a space that may not have any active flow movement. At the same time the volume of UCA must be reduced to prevent artefact from excessive signal, which can lead to loss of detail and posterior acoustic shadowing. In the experience of the authors the same concentration can be utilised regardless of whether a high frequency linear or curvilinear transducer is used. A further limiting factor is the ability to access the targeted cavity. Often this is via an invasive technique (e.g. nephrostomy, biliary drainage or catheterisation); the additional procedural risk may not be preferable. The invasive element and the recent development of endocavitary CEUS mean direct comparative studies with non-invasive techniques have not been performed in many areas such as with MR imaging. The dynamic nature of the studies involved, however, likens endocavitary CEUS more to fluoroscopy but without the need for a dedicated suite or use of radiation.

## Conclusion

Conventional CEUS is a technique gaining rapid popularity owing to its excellent safety profile, spatial and temporal resolution whilst also being portable and free of ionising radiation. Extrapolation of traditional intravascular use of CEUS allows for a range of endocavitary CEUS uses in diagnostic radiology particularly for the assessment of drain positions and evaluation of loculation, strictures and fistulae. The detailed resolution and real-time imaging also allow for myriad interventional uses of endocavitary CEUS.

Endocavitary CEUS is a novel technique and offers a dynamic modality assessment in real time, with operator discretion for visualisation of specified anatomy. The size of UCA microbubbles combined with both exquisite temporal and spatial resolution means it is ideally suited to endoluminal delineation with uses described above in the biliary and collecting systems as well as established intrauterine use and utility in interventional radiology. Experimental use for non-physiological cavities, vascular lines and bowel implementations have been performed at our institution and, although feasible, formal studies have not yet been conducted despite anecdotal benefit, as befits a new technique.
